# Phosphorylation of Rad9 at Serine 328 by Cyclin A-Cdk2 Triggers Apoptosis *via* Interfering Bcl-xL

**DOI:** 10.1371/journal.pone.0044923

**Published:** 2012-09-13

**Authors:** Zhuo Zhan, Kan He, Dan Zhu, Dan Jiang, Ying-Hui Huang, Yang Li, Chao Sun, Ying-Hua Jin

**Affiliations:** 1 Key Laboratory for Molecular Enzymology and Engineering of the Ministry of Education, College of Life Science, Jilin University, Changchun, China; 2 State Key Laboratory of Supramolecular Structure & Materials, Jilin University, Changchun, China; 3 Cancer Center, China-Japan Union Hospital, Jilin University, Changchun, China; Osaka University Graduate School of Medicine, Japan

## Abstract

Cyclin A-Cdk2, a cell cycle regulated Ser/Thr kinase, plays important roles in a variety of apoptoticprocesses. However, the mechanism of cyclin A-Cdk2 regulated apoptosis remains unclear. Here, we demonstrated that Rad9, a member of the BH3-only subfamily of Bcl-2 proteins, could be phosphorylated by cyclin A-Cdk2 *in vitro* and *in vivo*. Cyclin A-Cdk2 catalyzed the phosphorylation of Rad9 at serine 328 in HeLa cells during apoptosis induced by etoposide, an inhibitor of topoisomeraseII. The phosphorylation of Rad9 resulted in its translocation from the nucleus to the mitochondria and its interaction with Bcl-xL. The forced activation of cyclin A-Cdk2 in these cells by the overexpression of cyclin A,triggered Rad9 phosphorylation at serine 328 and thereby promoted the interaction of Rad9 with Bcl-xL and the subsequent initiation of the apoptotic program. The pro-apoptotic effects regulated by the cyclin A-Cdk2 complex were significantly lower in cells transfected with Rad9S328A, an expression vector that encodes a Rad9 mutant that is resistant to cyclin A-Cdk2 phosphorylation. These findings suggest that cyclin A-Cdk2 regulates apoptosis through a mechanism that involves Rad9phosphorylation.

## Introduction

Cyclin A-Cdk2, a Ser/Thr kinase, regulates mammalian cell cycle progression by phosphorylating specific substrates during S phase [1], [2]. There is increasing evidence that cyclin A-Cdk2 also plays an importantrole in apoptosis. Cdk2 activation has been observed inthe apoptosis ofultraviolet irradiatedmesangial cells [3], etoposide-treatedhuman leukemic cells [4], [5], growth factor-deprived HUVEC cells [6], ginsenoside-Rh2 (G-Rh2)-treated human hepatomacells [7], panaxadiol-treated SK-HEP-1 cells [8], and paclitaxel-treated HeLa cells [9]. We showed previously that Cdk2 activity, but notCdc2 activity, was selectively up-regulated and that this up-regulation wasrequired for the induction of apoptosis by G-Rh2 [7], panaxadiol [8], or etoposide [4] in several cancer celllines, including SK-HEP-1 cells and HeLa cells. Cyclin A-Cdk2 may act upstream of mitochondrial cytochrome *c* release in the apoptosis pathway [4], [9]. Although these studies suggest an essential role of cyclin A-Cdk2 in apoptosis, the precise molecular mechanism by which cyclin A-Cdk2 regulates apoptotic pathways is largelyunknown.

Studies of apoptosis suggest that Bcl-2 family proteins regulate the permeabilization of themitochondrial outer membrane (PMOM) and the release of cytochrome *c*. Anti-apoptotic Bcl-2family members function to block PMOM, whereas the multi-domain pro-apoptotic molecules Bax and Bak serve as obligatory effectors of cytochrome *c* release in response to diverse stimuli. The other subfamily, theBH3-only proteins (which contain only the BH3domain), can either interfere with the anti-apoptoticBcl-2 family membersor activate Bax andBak [10]–[14].

The Rad9 gene was firstly isolated from the fission yeast *Schizosaccharomycespombe*(*S. pombe*) by functional complementationof the radiosensitivity of corresponding mutant cells [15]. Later studies showed that the human Rad9 gene was evolutionarily conserved and played important roles in many fundamental biological processes, including the maintenance of genome stability, the control of cell cycle checkpoints, the promotion of resistance to DNA damage, andthe promotion of apoptosis [16]–[21]. Rad9 can bind to Rad1 and Hus1 to form a heterotrimeric complex (the 9-1-1 complex) [22]–[25], and it is believed to perform many of its surveillance activities as part of this 9-1-1 heterotrimer. Other studies have shown that Rad9 contains a Bcl-2 homology (BH3)-like domain that is typical of BH3-only pro-apoptotic family members and thatthe overexpression of Rad9 in a variety of human cell lines induces apoptosis [26], [27]. Rad9 phosphorylation at tyrosine 28 by c-Abl in U-937 cells is induced by UV irradiation, and the phosphorylation of Rad9 by protein kinase Cδ (PKCδ) enhances the association of Rad9 with Bcl-2 [28], [29].

Here, we show that Rad9 is a novel substrate for cyclin A-Cdk2under apoptotic conditions. Cyclin A-Cdk2 phosphorylated Rad9 at serine 328 in HeLa cells during apoptosis induced by the treatment of etoposide, an inhibitor of topoisomerase II. The phosphorylation of Rad9 promoted its translocation to mitochondria and enhanced its interaction with Bcl-xL. The ectopic expression of Rad9 and cyclin A dramatically increased Rad9 triggered apoptosis. Taken together, these findings suggest that Rad9 is a novel substrate for cyclin A-Cdk2 under apoptotic conditions and mediates apoptotic signaling from cyclin A-Cdk2 to the mitochondria.

## Results

### Rad9 is Phosphorylated by Cyclin A-Cdk2 in vitro and in vivo

**Figure 1:**
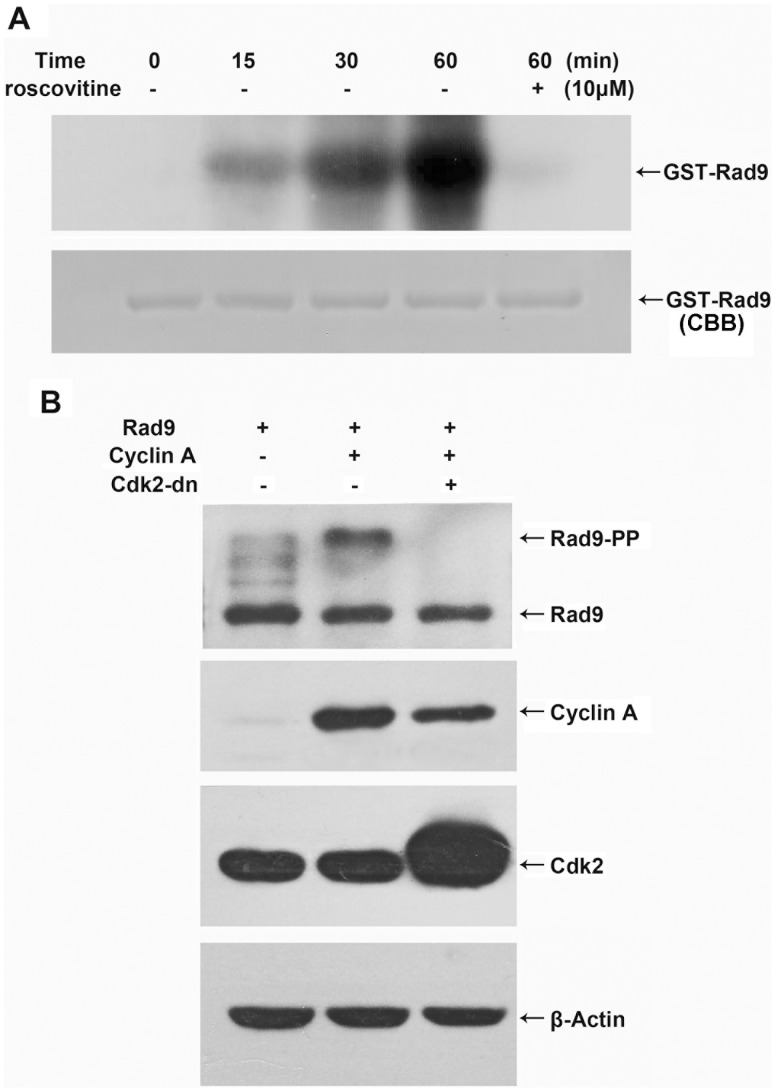
Cyclin A-Cdk2 phosphorylates Rad9 *in vitro* and *in vivo*. (A) The immunoprecipitatedcyclin A-Cdk2 complex was incubated with the GST-Rad9 fusion protein and/or roscovitine and [γ-^32^P] ATP at 30°C for the indicated times. The phosphorylated proteins wereresolved by SDS-PAGE stained with Coomassie brilliant blue (CBB), and detected by autoradiography. (B) HeLa cells were co-transfected with pCMV-cyclin A, pCMV-Cdk2-dn, and pCS4-Rad9. The lysates from the transfected cells were resolved by SDS-PAGE and analyzed by immunoblotting using antibodies against Rad9, cyclin A, Cdk2, and β-actin.

Previous studies have shown that cyclin A-Cdk2 activity is required for apoptosis induced by diverse stimuli [3]–[9]. Because cyclin A-Cdk2 is a protein kinase, we considered the possibility that it mightpromote apoptosis by directly phosphorylating and thereby activating a component of the cell death regulatory machinery. Rad9 is a phosphoprotein that can be phosphorylated by a number of kinases under specific conditions [30]. Rad9 is a BH3-only protein, and it can trigger apoptosis by inhibiting the anti-apoptotic proteins Bcl-2 and Bcl-xL [26]. To determine whether Rad9 is phosphorylated by cyclin A-Cdk2, we performed an *in vitro* kinase assay. Cyclin A-Cdk2 was immunoprecipitated using a Cdk2 antibody from etoposide-treated HeLa cells and induced strong phosphorylation of recombinant GST-Rad9 in a time-dependent manner;thisRad9phosphorylation was markedly reduced in the presence of roscovitine, an inhibitor of Cdk2(Fig.1A). To determine whetherRad9phosphorylationalso occurs in cells, HeLa cells were co-transfected with pCMV-Cyclin A or pCMV-Cdk2-dn with pCS4-Rad9, and Rad9phosphorylation wasassayed. We showed previously that the overexpression of cyclin A strongly activated Cdk2 in a dose-dependent manner and that the overexpression of Cdk2-dn inhibited Cdk2 activity [7].In the present study, Rad9phosphorylation status was characterized by analyzing its electrophoretic mobility. Immunoblotting analysis with ananti-Rad9 antibody showed that the slower-migrating (i.e., phosphorylated) form of Rad9 was clearly enhanced in cyclin A-transfected cells and disappeared completely in Cdk2-dn transfected cells (Fig. 1B). These findingsdemonstrate that cyclin A-Cdk2 phosphorylates Rad9 *in vitro* and *in vivo*.

### Rad9 is Phosphorylated by cyclin A-Cdk2 in Etoposide-treated HeLa Cells

**Figure 2:**
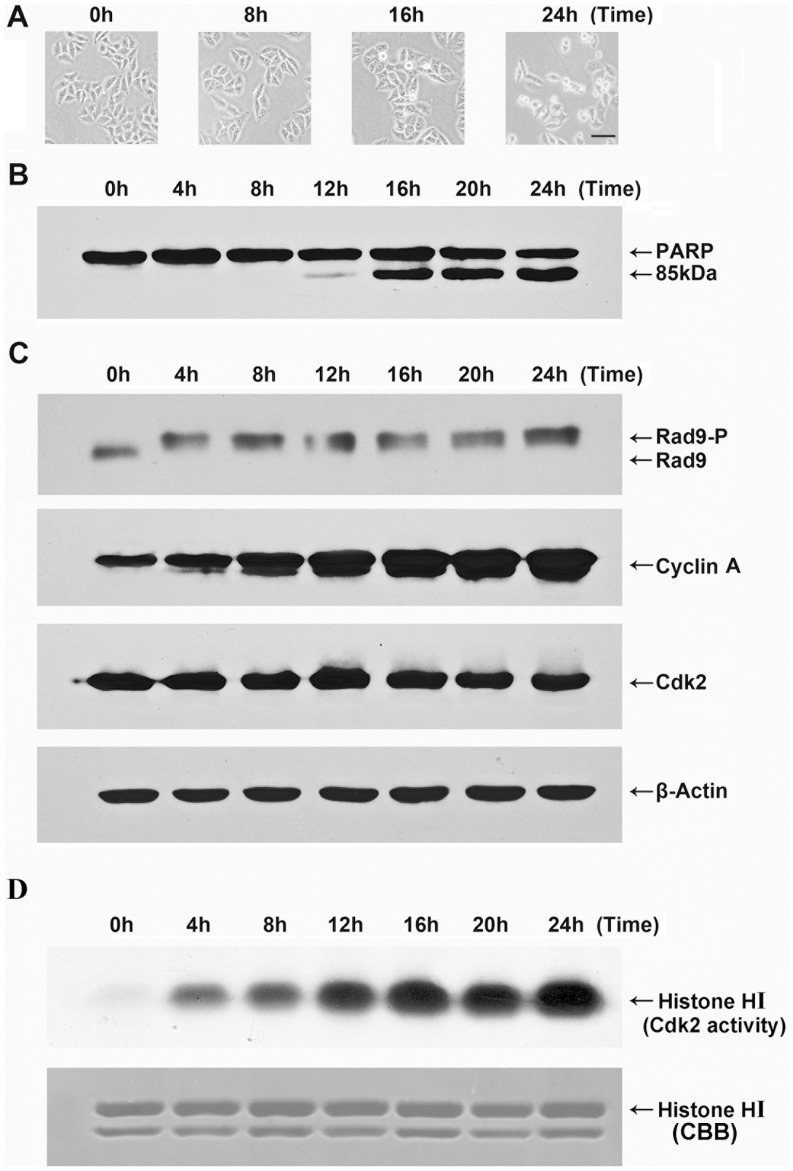
Phosphorylation of Rad9 duringetoposide-induced apoptosis. HeLa cells were treated with etoposide (50 µg/mL) for the indicated times. (A) The cells were imaged at 40×magnificationusing a light microscope. Bar, 50 µm. (B)Whole-cell extracts were resolved by SDS-PAGE and analyzed by immunoblotting with an anti-PARP antibody. (C) Whole cell extracts were resolved by 10% SDS-PAGE for 13 cm andanalyzed by immunoblotting using antibodies against Rad9, other extracts were resolved by 12% SDS-PAGE and analyzed by immunoblotting using antibodies againstCdk2, cyclin A, and β-actin. (D) Immunecomplex kinase assays for Cdk2 activity wereperformed using histone H1 as asubstrate, as described in theMaterials and methods.

Previouslystudy has shown that cyclin A-Cdk2 activity but not cdc2 activity was up-regulated during apoptosis induced by etoposide treatment [4]. To determine whether Rad9 is phosphorylated by cyclin A-Cdk2 in etoposide-treated apoptotic cells, we first examined the phosphorylation status of Rad9 by immunoblotting with anti-Rad9 antibodies. Apoptosis was monitored by examining cell morphology and caspase activity. Individual cells exhibitedseveral morphological changes that typically occur during apoptosis, such as cell rounding and membrane blebbing,after treatment for 16 h (Fig.2A). PARP, a well-known caspase-3 substrate, was cleaved to yieldan 85 kD fragment 16 h after the treatment of HeLa cells with etoposide (Fig. 2B). The electrophoretic mobility of Rad9 was clearly shifted beginning at 4 h post etoposide treatment, indicating thatRad9phosphorylation may haveoccurred at an early stage of etoposide-induced apoptosis in HeLa cells (Fig. 2C). The levels of cyclin A (Fig. 2C) and of cyclin A-Cdk2 activity (Fig. 2D) were up-regulated in a etoposide treating time-dependent manner.

**Figure 3:**
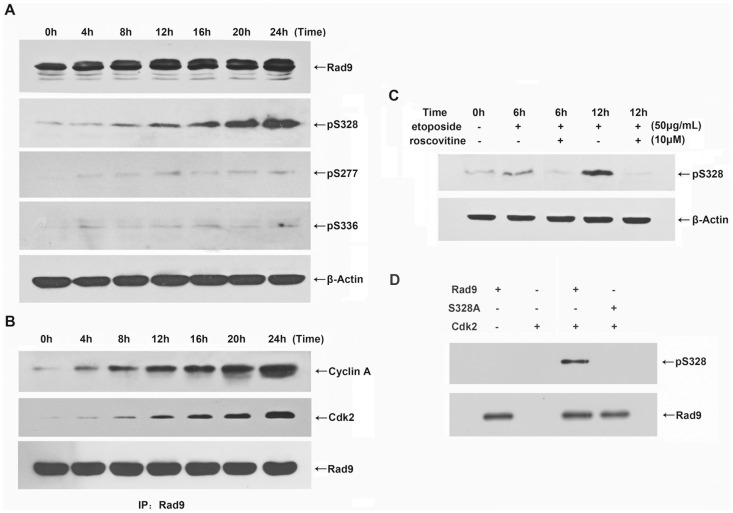
Cyclin A-Cdk2 promotes the phosphorylation of Rad9 serine 328 during etoposide-induced apoptosis in HeLa cells. HeLa cells were treated with etoposide (50 µg/mL) for the indicated times. (A) Whole-cell extracts were resolved by 12% SDS-PAGE and analyzed by immunoblotting using antibodies against phospho328-Rad9, phospho277-Rad9, phospho336-Rad9, and β-actin. (B) Lysates from treated cells were subjected to immunoprecipitation with anti-Rad9 antibody and immunoblotting with antibodies against cyclin A, Cdk2, and Rad9. (C) The cells were treated with etoposide (50 µg/mL) for the indicated times, and roscovitine (10 µM) was added to the medium 1 h before the etoposidetreatment. The cell lysates were analyzed by immunoblotting with antibodies against phospho328-Rad9 and β-actin. (D) The immunoprecipitatedcyclin A-Cdk2 complex was incubated with the GST-Rad9 or GST-Rad9-S328A fusion protein and ATP at 30°C for 30 min. The proteins were resolved by SDS-PAGE and analyzed by immunoblotting using antibodies against phospho328-Rad9, and Rad9.

Next, we examined whether cyclin A-Cdk2 phosphorylates Rad9 in etoposide-treated HeLa cells. Immunoblot analyses of immunocomplexes that were immunoprecipitated from HeLa cells by specific anti-Rad9 antibody demonstrated that both cyclin A and Cdk2 interacted with Rad9 after the cells were treated with etoposide for 8 h (Fig. 3B). To identify the phosphorylation site within Rad9, we performedimmunoblotting analyses using phospho-specific antibodies againstphospho328-Rad9, phospho277-Rad9, and phospho336-Rad9. The phosphorylation of serine 328 was appeared 8 h after etoposide treatment and significantly increased in a time-dependent manner, and the timing ofthis increase was consistent with that of the interaction between cyclin A and Cdk2 (Fig. 3A, B). In addition, the etoposide-induced phosphorylation of Rad9 at serine 328 was completely inhibited in HeLa cells that were co-treated with roscovitine (Fig.3C). However,serine 277 and serine 336 showed only moderate phosphorylation in etoposide-treated cells (Fig.3A).Toprovide further evidence that serine 328 of Rad9 can be phosphorylated by cyclin A-Cdk2, we performed an *in vitro* kinase assay. Cyclin A-Cdk2 was immunoprecipitated using a Cdk2 antibody from etoposide-treated HeLa cells and incubated with recombinant GST-Rad9 or GST-Rad9-S328A, a mutant version of Rad9, which is resistant to phosphorylation at serine 328. Immunoblotting analyses using phospho-specific antibodies againstphospho328-Rad9 showed that Rad9was phosphorylated at serine 328 and this phosphorylation was disappearedin the Rad9-S328A (Fig. 3D). These findingsdemonstrate that cyclin A-Cdk2 phosphorylates Rad9 at serine 328 inetoposide-treatedapoptotic HeLa cells.

**Figure 4:**
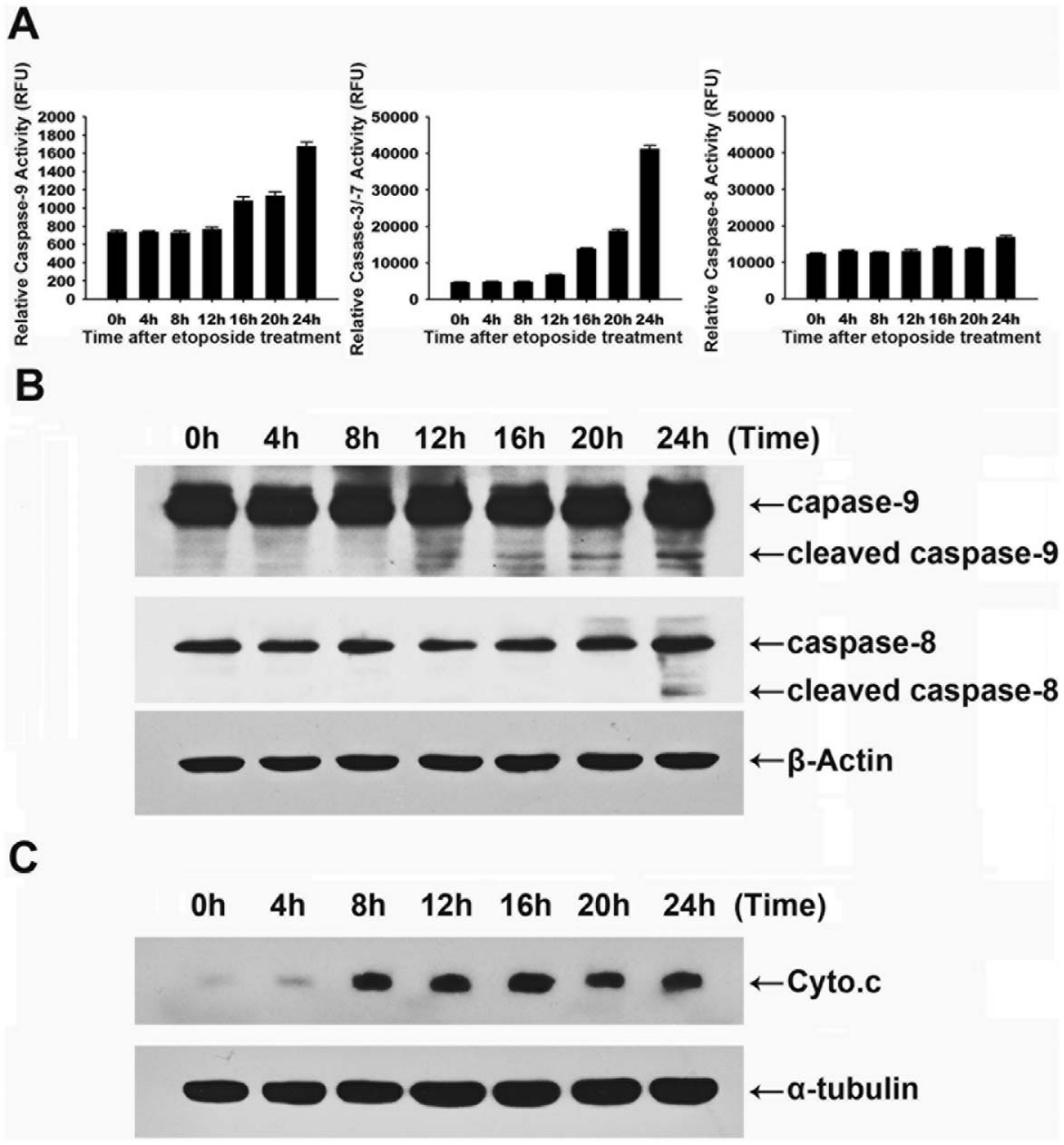
Etoposide induces apoptosis through caspase-9 and caspase-3 activation, mediated by mitochondrial cytochrome *c* release. HeLa cells were treated with etoposide (50 µg/mL) for the indicated times. (A) Cell-free caspase-3, -8, and -9 activities were analyzed using specific substrates (Ac-DEVD-AFC, Ac-IETD-AFC, and Ac-LEHD-AFC, respectively). (B) The cells were analyzed by immunoblotting for caspase-8 and caspase-9. (C) Equal amounts of proteins from the cytosolic fraction were resolved by SDS-PAGE and analyzed by immunoblotting using antibodies against cytochrome *c* and α-tubulin.

To determine the functional effect of the cyclin A-Cdk2-induced phosphorylation of Rad9 at serine 328 in HeLa cell apoptosis, we first investigated the caspase activation pathway in etoposide-treatedHeLa cells. The activation kinetics of the caspases revealed that the activities of initiator caspase-9 and effector caspase-3/−7 were up-regulated in cells treated with etoposide for 12 h, whereas caspase-8 activity remained unchanged until 24 h of treatment (Fig.4A). The immunoblotting analysis showed that caspase-9 was cleaved to yield catalytically active forms after 12 h, whereas caspase-8cleavage occurred after 24 h (Fig.4B). Thus, the caspase cascade was initiated by the proteolytic activation of the initiator caspase-9 but not of the initiator caspase-8 in the process of etoposide-induced apoptosis in HeLa cells.Further study of the apoptosis pathway showed that cytochrome*c*was released from the mitochondria to the cytosol after8h ofetoposide treatment (Fig.4C). These findings indicate that etoposide induces HeLa cell apoptosis througha mitochondria-mediated caspase-9 activation pathway.

### The Phosphorylation ofserine 328 up-regulates Rad9 Translocation from the Nucleus to the Mitochondria

**Figure 5:**
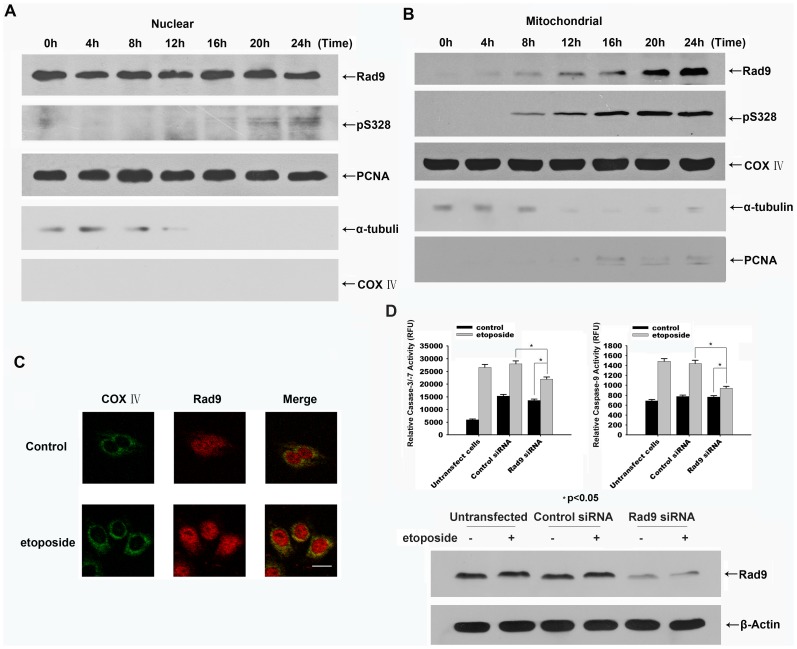
Serine 328-phosphorylated Rad9 is translocated from the nucleus to the mitochondria during etoposide-induced apoptosis in HeLa cells. HeLa cells were treated with etoposide (50 µg/mL) for the indicated times. Equal amounts of protein from the nuclear fractions (A), and mitochondrial fractions (B) were resolved by SDS-PAGE and analyzed by immunoblotting using antibodies against Rad9, phospho328-Rad9, PCNA, α-tubulin, and COX IV. (C) The cells were treated with etoposide(50 µg/mL) for 20 h. The cells were fixed and stained with anti-Rad9 and anti-COX IV antibodies and analyzed by confocal microscopy using appropriate filters for the visualization of green, red, or combined fluorescence resulting from the presence of FITC and rhodamine molecules. Bar, 20 µm. (D) HeLa cells were transfected with negative control or Rad9 siRNA followed by the treatment with etoposide(50 µg/mL) for 20 h. Top: Cell extracts were assayed for caspase-3 and caspase-9 activities using the specific substrates Ac-IETD-AFC and Ac-DEVD-AFC (* p<0.05). Bottom: The contents of Rad9 and Actin in cell lysates were examined by immunoblotting.

To determine whetherRad9 phosphorylation regulates the mitochondria-mediated activation of caspase-9, we examined the distribution of Rad9 and of serine 328-phosphorylated Rad9 in etoposide-treated HeLa cells. Immunoblot analysis showed that Rad9 was translocated from the nucleus to the mitochondria in cells that were treated for 8 h. The serine 328-phosphorylated form of Rad9 appeared in the mitochondrial fraction after 8 h of treatment and gradually increased afterwards (Fig.5A, B). Immunofluorescence analysis also showed that Rad9 was translocated from the nucleus to mitochondria in etoposide-treated HeLa cells (Fig. 5C). The timing of Rad9 phosphorylation at serine 328 upon etoposide treatment coincided well with that of the interaction of Rad9 with cyclin A-Cdk2 and that of the occurrence of mitochondrial Serine328 phosphorylated Rad9 in HeLa cells. These findings suggest that the phosphorylation of Rad9 at serine 328 promotesthe translocation of Rad9 from the nucleus to mitochondria in etoposide-treated HeLa cells.

To provide further evidence for the proapoptosis function of Rad9 we silenced its expression in HeLa cells. The specific inhibition of Rad9 using RNAi technology largely reduced the activation of caspase-3/−7 and caspase-9 in etoposide-treated HeLa cells (Fig 5D). Taken together, these results suggest that the cyclin A-Cdk2-mediated phosphorylation of Rad9 is crucial for the apoptosis progression.

### Cyclin A-Cdk2-catalyzed Phosphorylation of Rad9 Promotes Apoptosis

**Figure 6:**
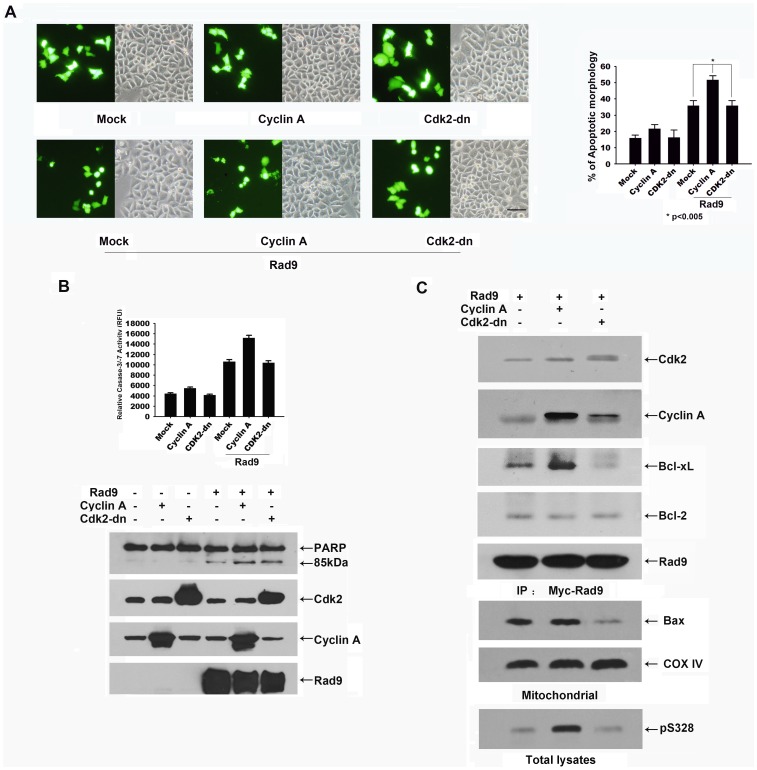
Cyclin A-Cdk2-induced phosphorylation of Rad9 promotes Rad9-mediated apoptosis. HeLa cells were co-transfected with pCMV-GFP, pCS4-myc-Rad9, pCMV-Cyclin A, or pCMV-Cdk2-dn. (A) Left: Bright-field and GFP fluorescence of the same field (×100) of transfected cells. (Top: HeLa cells were co-transfected with pCMV-GFP and pCMV-Cyclin A or pCMV-Cdk2-dn; bottom: HeLa cells were co-transfected with pCMV-GFP, pCS4-myc-Rad9, pCMV-Cyclin A, or pCMV-Cdk2-dn.) Right: GFP-expressing cells with blebbing or normal morphology were counted. The extent of apoptosis was determined by counting GFP-expressing cells with blebbing or normal morphology in three randomly selected fields (80–100 cells per field). The average numbers in three different fields from two independent experiments are shown(* p<0.005). Bar, 50 µm. (B) Caspase-3 activity in transfected cells was determined using Ac-DVED-AFC as a substrate. The levels of PARP, Cdk2, cyclinA, and Rad9were analyzed by immunoblotting. (C) The lysates of transfected cells were immunoprecipitatedwith an anti-myc antibody and then immunoblotted with antibodies against Cdk2, cyclin A, Bcl-2, and Bcl-xL, or immunoblot analysis of Bax and COX IV in mitochondrial, and phospho328-Rad9 in the total lysates.

To test whether the phosphorylation of Rad9 by cyclin A-Cdk2 promotes apoptosis, HeLa cells were co-transfected with pCMV-GFP, pCS4-Rad9 and pCMV-Cyclin A or pCMV-Cdk2-dn. The transfected cells were distinguished from untransfected cells on the basis of GFP expression. At 24 h after transfection, cell morphology was examined in the transfected cells.Approximately 35% of the Rad9-transfected cellsdisplayed apoptotic morphology (including cell rounding and membrane blebbing), and 51% of the cells co-transfected with Rad9 and cyclin A displayed this morphology. In contrast, only 15–20% of thecyclin A-transfected cells exhibited apoptotic morphology (Fig.6A). We examined the activity of effector caspase-3/−7 and the cleavage of PARP under the same experimental conditions. Caspase-3/−7 activity was elevated in the Rad9-transfected cellsand further elevated in the cells co-transfected with Rad9 and cyclin A. The immunoblotting analysis showed that PARP cleavage occurredin theRad9-transfected cells and was enhanced in the cellsco-transfected with cyclin A and Rad9 (Fig.6B).

Rad9 has been reported to contain a BH3-like region that can interact with the anti-apoptotic Bcl-2 family proteins, Bcl-2 or Bcl-xL, and thereby promote apoptosis [26]. To determine whether the phosphorylation of Rad9 at serine 328 by cyclin A-Cdk2 regulates the interaction between Rad9 and Bcl-2 or Bcl-xL, we co-transfected HeLa cells with pCS4-myc-Rad9 andpCMV-Cyclin A or pCMV-Cdk2-dn and examined the interaction between Rad9 and Bcl-2 or Bcl-xL. After 36 h of transfection, the cells wereimmunoprecipitated with an anti-mycantibody,and the immunoprecipitatewas examined by immunoblot analysis with antibodies against Cdk2, cyclin A, Bcl-2, and Bcl-xL. Rad9 was found tointeractwith Cdk2, cyclin A, Bcl-2, andBcl-xL. The amount of Bcl-xL that was associated with the Rad9 complex was significantly increased in cyclin Aco-transfected cells and was lower in Cdk2-dnco-transfected cells than inthe cells transfected with Rad9 alone (Fig.6C). Immunoblot analysis showed that the phosphorylation of Rad9 at serine 328 was dramatically increased in cyclin A co-transfected cells, and nearly disappeared in Cdk2-dn cotransfected cells (Fig. 6C). Next, we examined mitochondrial Bax translocation in Rad9 overexpressed HeLa cells. As shown in Fig. 6C, we found that Bax significantly translocated to mitochondria in Cyclin A co-transfected HeLa cells, whereas this translocation was dramatically reduced by Cdk2-dn transfection (Fig. 6C). Taken together, these results suggest that the cyclin A-Cdk2-mediated phosphorylation of Rad9 is crucial for the interaction of Rad9 with Bcl-xL and subsequent apoptosis progression.

### Rad9S328A Promotes Apoptosis Less Efficiently than does Rad9

**Figure 7:**
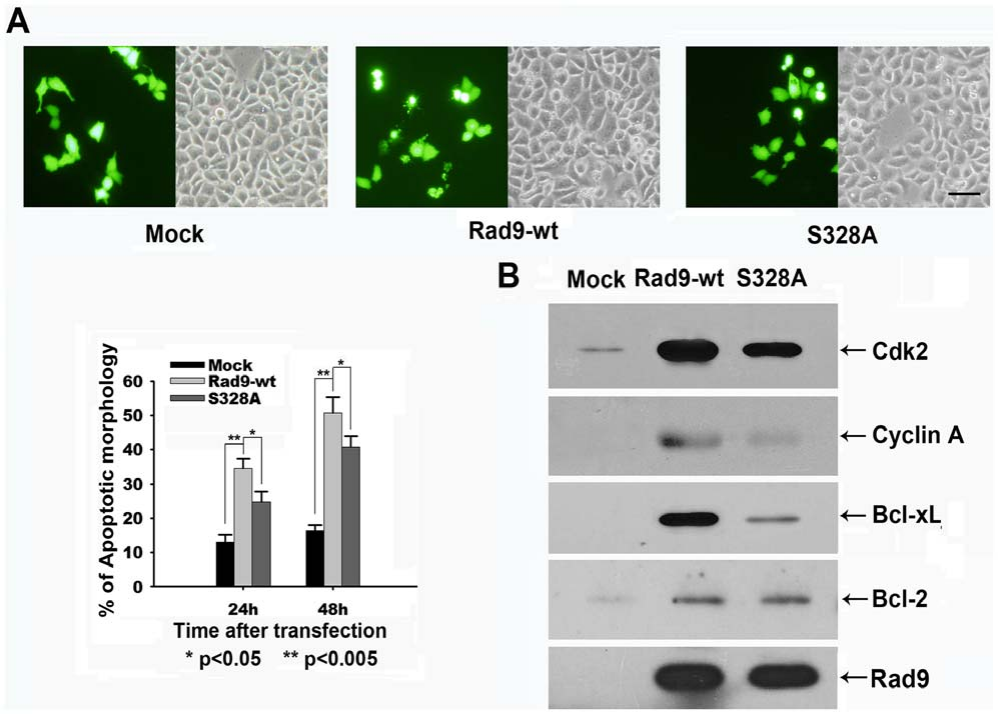
Rad9S328A promotes apoptosis less efficiently than does Rad9. HeLa cells were co-transfected with pCS4, pCS4-Myc-Rad9-wt, pCS4-Myc-Rad9-S328A, and pCMV-GFP. (A) Top: Bright-field and GFP fluorescence of the same field (×100) of transfected cells. Bottom: GFP-expressing cells with blebbing or normal morphology were counted at the indicated times. The average numbers in three different fields from two independent experiments are shown (* p<0.05, ** p<0.005). Bar, 50 µm. (B) The lysates of transfected cells were immunoprecipitated with the anti-myc antibody and then immunoblotted with antibodies against Cdk2, cyclin A, Bcl-2,and Bcl-xL.

To test the hypothesis that the phosphorylation of Rad9 at serine 328 promotes its pro-apoptotic activities in HeLa cells, we examined the effect ofthe ectopic expression of Rad9S328A, a mutant version of Rad9 that is resistant to cyclin A-Cdk2 phosphorylation, on the induction of apoptosis. Apoptotic morphologywas observed in 33% and 50% of the Rad9-transfected cells but in only 23% and 40% of the Rad9S328A-transfected cellsat 24 h and 48 h post transfection, respectively (Fig.7A). Finally, we tested whether Rad9 phosphorylation at serine 328 regulates the interaction of Rad9 with Bcl-2 or Bcl-xL under the same experimental conditions. The immunoblot analysis ofimmunocomplexes that wereimmunoprecipitated with anti-myc antibody showedthat thedisphosphorylation of Rad9 at serine 328 greatly reduced the interaction of Rad9 with Bcl-xL but not with Bcl-2 (Fig. 7B). Taken together, these findings suggest that cyclin A-Cdk2 regulates Rad9-mediated apoptosis by phosphorylatingRad9 at serine 328 in HeLa cells.

## Discussion

Four major observations were made in this study: (i) Rad9, a member of the BH3-only protein subfamily, can be phosphorylated by cyclin A-Cdk2 *in vitro* and *in vivo*; (ii) cyclin A-Cdk2 phosphorylates Rad9 at serine 328 during etoposide-induced apoptosis in HeLa cells; (iii) the up-regulation of cyclin A-Cdk2 activity enhances Rad9-induced apoptosis by phosphorylating Rad9 at serine 328; and (iv) the phosphorylation of Rad9 at serine 328 is required for the interaction of Rad9 with Bcl-xL.

Rad9 contains a BH3-like region and promotes apoptosis in ara-C-treated U937 cells [28], [29]. Previous studieshave shown that cyclin A-Cdk2, a cell cycle-regulated protein kinase,canbe activated by various stimuli and plays key roles in apoptotic processes[3]–[9]. We investigated whether cyclin A-Cdk2 phosphorylates Rad9 during apoptosis. We chose to use etoposide-induced apoptosis in HeLa cells as an experimental system because cyclin A-Cdk2, but not cyclin B-Cdc2, is selectively activated in this process, and this activation is essential for the progression of apoptosis at an early stage [4]. The intracellular levels of cyclin A (Fig. 2C), the activation of cyclin A-Cdk2 (Fig. 2D), and the electrophoretic mobility shift of Rad9 were all upregulated in etoposide treated HeLa cells. In addition, the coimmunoprecipitation analysis showed that Rad9 interacted with cyclin A and Cdk2 ina time-dependent manner following etoposide treatment (Fig. 3B). These findings suggest that the selective up-regulation of cyclin A-Cdk2 may beassociated with Rad9 phosphorylation during etoposide-induced apoptosis in HeLa cells.

Since Rad9 is a checkpoint protein, it has been shown to be phosphorylated by several kinase including ataxia-telangiectasia-mutated (ATM), PKCδ and c-Abl upon genotoxic stimuli [28], [29], [31]. Interestingly in our study, the phosphorylation form of Rad9 was observed as early as 4 h after etoposide treatment (Fig 2C), while the interaction between Cdk2 with Rad9 was occurred after 8 h treatment (Fig. 3B) in HeLa cells. We then suggest that the early stage (at 4h) phosphorylation of Rad9 maybe not driven by cyclin A-Cdk2.

Rad9 contains nine potential consensus Cdk2 phosphorylation sequences (S/TPXK/R) [32], but an *in vitro* kinase assay showed that only three potentialphosphorylation sites (serine 277, serine 328, and serine 336) were phosphorylated by cyclin A-Cdk2 (data not shown). We analyzed the phosphorylation at these three serine residuesin etoposide-treated HeLa cells using phospho-specific antibodies. The phosphorylation of Rad9 at serine 328 was observed at 8h after the etoposide treatment and subsequentlyincreased significantly in a time-dependent manner. In contrast, serine 277 andserine 336 of Rad9 were only moderately phosphorylated under the same experimental conditions (Fig. 3A). The timing of Rad9 phosphorylation at serine 328 upon etoposide treatment coincided well with that of the interaction of Rad9 with cyclin A-Cdk2. Furthermore, the phosphorylation of Rad9 at serine 328 was blocked bythe addition of roscovitine to the culture medium (Fig. 3C). These findings indicate thata specifically activated cyclin A-Cdk2 kinase phosphorylates endogenous Rad9 at serine 328 during etoposide-induced apoptosis in HeLa cells.

A previous study suggested that etoposide-induced apoptosis in head and neck carcinoma cellsis dependent on mitochondria-mediated caspase-9 activation [33]. Similarly, we observed that caspase-9, but not caspase-8,is activated in the early stages of etoposide-induced apoptosis in HeLa cells (Fig. 4A, B). Consistently with this observation, cytochrome *c*was released from mitochondria to the cytosol 8h after etoposide treatment (Fig. 4C), indicating that etoposide-induced apoptosis in HeLa cellsis initiated by a mitochondrial pathway.

A previous report showed that the overexpression of Rad9 induces apoptosis and that this apoptosis can be blocked by Bcl-2 or Bcl-xL [26]. In the present study, cell fractionation and immunofluorescence analyses showed that Rad9 translocated from the nucleus to the mitochondria in etoposide-treated HeLa cells (Fig. 5A–C). Almost all of the serine 328-phosphorylated Rad9 was found in the mitochondria, suggesting thatRad9 phosphorylationat serine 328 is associated withthe pro-apoptotic function of Rad9 in HeLa cells. Importantly, the phosphorylation of Rad9 at serine 328, the translocation of Rad9 to mitochondria, and the cytochrome *c* release occurred at the same time period in etoposide treated HeLa cells. In addition, the coimmunoprecipitation analysis showed that Rad9 interacted with Bcl-xL in a time-dependent manner following etoposide treatment (Fig. S1). We thereforehypothesized that Rad9 phosphorylation at serine 328 might regulate the caspase-9/−3 activation pathway that was initiated by mitochondrial cytochrome *c release*.

Rad9-induced apoptosis was significantly (p<0.005) enhanced by the elevation of cyclin A-Cdk2activitythroughthe overexpression of cyclin A (Fig. 6A, B).The phosphorylation of Rad9 at serine 328 and the interaction between Rad9 with Bcl-xL were dramatically increased by cyclin A overexpression and completelyinhibited by Cdk2-dn overexpression (Fig. 6C). In addition, the pro-apoptotic activity of Rad9S328A was significantly lower than that of wild-type Rad9 (Fig. 7A). The amount of Bcl-xL associated with the Rad9 complex was much lower in the Rad9S328A-transfected cells than in the Rad9-transfected cells. Interestingly, the amount of Bcl-2 associated with the Rad9 complex was almost equal in these two backgrounds (Fig. 7B). All these findings indicate that cyclin A-Cdk2 induced phosphorylation of Rad9 at serine 328 promotes theinteracting with Bcl-xL, but not Bcl-2, and thereby triggers the apoptotic progress.

Cyclin A overexpression enhanced the apoptosis in the presence of Rad9-S328A, but still less than that in presence of Rad9-wt (Fig. S2). We suggested that there might be other phosphorylation sites involved in the pro-apoptotic function of Rad9. In addition, part of overexpressedRad9-wt and Rad9-S328A translocated to mitochondrial, and the amount of Rad9-S328A was much less than that of Rad9-wt (Fig. S3).The partial suppression of Rad9-induced apoptosis by Cdk2-dn and the remaining pro-apoptotic activity of Rad9S328A observed in present study (Fig 6 and 7) may result from the interaction between the serine 328 dephosphorylated form of Rad9 and Bcl-2. These possibilities will be examined in future studies.

It will also be important to determine whether cyclin A-Cdk2 directly activates other apoptosis regulatory proteins in a phosphorylation-dependent manner and whether serine 328 of Rad9 is the target of other protein kinases that transduce apoptotic signals. In this study, we observed that the phosphorylation of Rad9 at serine 328 triggers the interaction of Rad9 with Bcl-xL. The conformational changes upon this phosphorylation and the consequent interaction activity of Rad9 will be studied by structure analysis in future.

To provide further evidence for the apoptosis mediating activity of endogenous Rad9, we silenced its expressions using RNAi technology. The caspase-cascade was largely inhibited in Rad9 down regulated cells upon etoposide treatment (Fig. 5D).

The Bcl-2 family member Baxtranslocates from thecytosol to mitochondria, where itoligomerizes andpermeabilizes the mitochondrial outer membraneto promote apoptosis [34], [35].Previous study has shown that Cdk2 activity is involved in the mitochondrial translocation of Bax in etoposide-induced HeLa cell apoptosis [4]. In present study, we also observed that Bax translocated to mitochondria in Rad9-induced apoptosis of HeLa cells, and this translocation was dramatically inhibited by overexpression of Cdk2-dn (Fig. 6C). Previous study has shown that Bcl-xLretrotranslocatesBaxfrom the mitochondria into the cytosol in normal cells [36], thus, we suggested that the interaction of Rad9 with Bcl-xL might enhance the mitochondrial translocation of Bax by disturbing Bcl-xL-mediated Baxretrotranslocation in Rad9 overexpressed HeLa cells.

The present findings suggest that Rad9 is a novel substrate of cyclin A-Cdk2 and that it is phosphorylated at serine 328 during etoposide-induced apoptosis in HeLa cells.The phosphorylation of Rad9 at serine 328 is important for Rad9’s functions, includingits translocation from the nucleus to the mitochondria, its interaction with Bcl-xL, and itsconsequent pro-apoptotic activity.

## Materials and Methods

### Materials

Etoposide and roscovitine were purchased from Sigma (St. Louis, MO).[γ-^32^P]ATP was purchasedfrom Amersham Pharmacia Biotech. The caspase substrates Ac-DVED-AFC, Ac-IETD-AFC, and Ac-LEHD-AFC were purchased from Calbiochem (Darmstadt, Germany). The Mitochondria Isolation Kit was purchased from Pierce (Rockford, IL). Antibodies against poly(ADP-ribose) polymerase (PARP), Cdk2, cyclin A, PCNA, cytochrome *c*, α-tubulin, and β-actin were purchased from Santa Cruz Biotechnology(Santa Cruz, CA). Antibodies against caspase-8, caspase-9, Bcl-xL, Bcl-2, and Cox IV were purchased from Cell Signaling Technology (Danvers, MA). The antibody against Rad9 was purchased from Abcam (Cambridge, UK). Antibodies against pS328, pS277, and pS336 were purchased from Abnova (Walnut, CA). All other drugs and chemicals were purchased from Sigma.

### Methods

#### Cell culture and etoposide treatment

HeLa cells were maintained as monolayer cultures in Dulbecco’s modified Eagle’s medium (DMEM) supplemented with 10% (*v/v*) heat-inactivated calf serum, 100 U/mL penicillin, and 100 µg/mL streptomycin. Etoposide was added at a final concentration of 50 µg/mL to log phase cell cultures for the indicated time periods. Both floating and adherent cells were harvested for immunoblotting and caspase activity assays.

#### Preparation of total protein in cell lysates

The cells were washed with ice-cold PBS and solubilized in a lysis buffer containing 20 mMTris (pH 7.5), 0.5% Triton X-100, 2 mM MgCl_2_, 1 mMDTT, 1 mMEGTA, 50 mM b-glycerol phosphate, 25 mMNaF, 1 mM Na_3_VO_4_, 2 mg/mL leupeptin, 2 mg/mL pepstatin A, 2 mg/mL antipain, and 1 mMPMSF. After incubation on ice for 1 h, the insoluble materials were removed by centrifugation at 12,000×*g* for 15 min, and the supernatants of the cell lysates contained the total protein.

#### Transient transfection analysis of apoptosis in transfected cells

HeLa cells were prepared and transfected using PolyFect Transfection Reagent (Qiagen, Valencia, CA) according to the manufacturer’s instructions. In the experiments shown in Fig. 1B, HeLa cells were co-transfected with 0.5 µg pCS4-Rad9 and 1.5 µgpCMV-Cyclin A and/or 0.5 µg pCMV-cdk2-dn per well in 6-well plates. In the experiments shown in Fig. 6, HeLa cells were transfected with 0.3 µgpCMV-GFP and 0.6 µgpCMV-Cyclin A or 0.6 µg pCMV-cdk2-dn and/or 1.2 µg pCS4-Rad9 per well. In the experiments shown in Fig. 7, each well was transfected with 0.5 µgpCMV-GFP and 1.5 µg pCS4-Rad9-wt or 1.5 µg pCS4-Rad9-S328A. After transfection for 24, 36, or 48 h, the cells were examined by fluorescence microscopy (Olympus, Tokyo, Japan). The extent of apoptosis was determined by counting GFP-expressing cells with blebbing or normal morphology in 3 randomly selected fields (80–100 cells per field).

#### Caspase assay

The cell-free caspase assay was performed by incubating 50 µg of cell lysate with 200 nM Ac-DEVD-AFC (for caspase-3), Ac-IETD-AFC (for caspase-8), or Ac-LEHD-AFC (for caspase-9) in a reaction buffer containing 20 mMHEPES, pH 7.4, 100 mMNaCl, 10 mMDTT, 0.1% CHAPS, and 10% sucrose at 37°C for 1 h. The reaction was monitored by fluorescence emission at 505 nm and excitation at 405 nm.

#### Preparation of subcellular fractions

Nuclear protein extracts were prepared as described previously [4]. The cells were resuspended in homogenization buffer (250 mM sucrose, 20 mMHEPES, 10 mMKCl, 1.5 mM MgCl_2_, 0.1 mM EDTA, 1 mM EGTA, 1 mM DTT, and 0.1 mM PMSF, pH 7.5). The cells were then homogenized with a Dounce homogenizer, and the homogenates were centrifuged at 4°C and 1000×*g* for 5 min to pellet the nuclear fraction. Mitochondria extracts were prepared using the Mitochondria Isolation Kit (Pierce) for cultured cellsaccording to the manufacturer’s instructions. Isolated nuclei and mitochondria were solubilized in a lysis buffer containing 20 mMTris-HCl (pH 7.5), 150 mMNaCl, 1% NP-40, 0.5% deoxycholate, 0.1% SDS, 2 mM MgCl_2_, 1 mMDTT, 1 mMEGTA, 50 mM b-glycerol phosphate, 25 mMNaF, 1 mM Na_3_VO_4_, 2 mg/mL leupeptin, 2 mg/mL pepstatin A, 2 mg/mL antipain, and 1 mM PMSF. After incubation on ice for 1 h, the insoluble materials were removed by centrifugation at 12,000×*g* for 15 min, and the supernatants of the nuclear and mitochondrial extracts contained the total protein.

#### Immunoblot analysis

An aliquot (50 µg protein) of each sample was resolved by SDS-polyacrylamide gel electrophoresis (SDS-PAGE) and electro-transferred onto a polyvinylidenedifluoride (PVDF) membrane (Millipore, Bedford, MA). The membrane was blocked with 5% nonfat milk and probed with a specific primary antibody. The membrane was then washed and incubated with horseradish peroxidase-coupled anti-mouse or anti-rabbit IgG, and the protein bands were visualized by enhanced chemiluminescence (ECL, Amersham Biosciences, Piscataway, NJ).

#### Expression and purification of the GST-Rad9 fusion protein

pGEX-4T-1-Rad9 was transformed into *E. coli* BL21. The transformed *E. coli* were cultured in 200 mL LB medium containing 100 µg/mL ampicillin at 37°C until the A_600_ reached 0.5, and then protein expression was induced by adding isopropyl β-D-1-Thiogalactopyranoside(IPTG) to a final concentration of 0.2 mM at 25°C for 16 h. The GST-Rad9 fusion protein was purified using a GST-glutathione affinity system (GE) according to the manufacturer's instructions. Purified GST-Rad9 was used as the substrate for the *in vitro* kinase assay.

#### Immunoprecipitation and *in vitro* kinase assay

An aliquot (200 µg protein) of each cell extract was precleared with protein A-agarose beads, and the supernatant was incubated with anti-Cdk2 antibody for 4 h. The Cdk2 immunocomplexeswere collected after incubation with protein A-agarose beads for 2 h. The immunocomplexes were washed three times with immunoprecipitationlysis buffer and twice with kinase assay buffer containing 50 mMTris pH 7.5, 10 mM MgCl_2_, 1 mMdithiothreitol, 1 mMEGTA, 50 mM β-glycerol phosphate, 25 mMNaF, 0.1 mM Na_3_VO_4_, 1 µg/mL leupeptin, 1 µg/mL pepstatin A, 1 µg/mL antipain, and 1 mMPMSF. The immunocomplexes were then incubated for 15, 30, or 60 min at 30°C in 50 µL of the kinase assay buffer supplemented with purified recombinant GST-Rad9 (Fig. 1A) or 1 µg of histone H1 (Upstate Biotechnology) (Fig. 2D), 10 µCi [γ-^32^P] ATP (10 µM), 5 mmol/L protein kinase A inhibitor, and 20 mmol/L EGTA. The reaction was resolved by 12% SDS-PAGE, and the Cdk2 kinase activities were determined by autoradiography.

#### Immunofluorescence

The cells were cultured overnight on sterile glass cover slips in 6-well plates. The cells were treated with etoposide for 20 h to induce apoptosis and subsequently fixed with 4% formaldehyde for 10 min, rinsed three times with PBS, and permeabilized with 0.1% Triton X-100 for 15 min. The cells were again rinsed three times with PBS, blocked for 30 min with blocking solution (5% BSA in 0.1% Triton X-100), and then rinsed three times with blocking solution. The cells were incubated for 3 h with primary antibodies specific to each target, including rabbit polyclonal anti-Rad9 (1∶200) and mouse monoclonal anti-COX IV (1∶200). The cells were rinsed three times in blocking solution and incubated in secondary antibody (FITC-conjugated goat anti-rabbit secondary antibody, 1∶300; rhodamine-conjugated rabbit anti-mouse secondary antibody, 1∶300). The cells were washed twice in PBS and then mounted by inverting onto mounting medium on glass slides. The slides were stored at 4°C and analyzed by fluorescence confocal microscopy (Olympus).

### RNA Interference

3×10^5^ HeLa cells in six-well dishes were transfectedwith a final concentration of 50 nM small interfering RNA (siRNA) duplexes using HiPerFect Transfection Reagent (Qiagen, Valencia, CA), according to the manufacturer’s instruction. siRNA duplexes for Rad9 and controlled siRNA were from Bioneer Co. (Daejeon, Korea). siRNA sense sequences were shown as follows: the siRNA sequence targeting human Rad9, 5′-GAAUUCUAAGAGCCUUGGA(dTdT)-3′; the negative control sequence, 5′-CCUACGCCAAUUUCGU(dTdT)-3′. After incubation for 24 h, cells were treated with etoposide (50 µg/mL) for 20 h. Finally, the total protein in cell lysates was prepared for caspase-3, caspase-9 activities assay and immunoblotting analysis of Rad9 and β-Actin.

## Supporting Information

Figure S1 HeLa cells were treated with etoposide (50 µg/mL) for the indicated times. Lysates from treated cells were subjected to immunoprecipitation with anti-Rad9 antibody and immunoblotting with antibodies against Bcl-xL and Rad9.
(TIF)

Figure S2 HeLa cells were co-transfected with pCMV-GFP, pCS4-myc-Rad9-S328A, pCMV-Cyclin A, or pCMV-Cdk2-dn. Top: Bright-field and GFP fluorescence of the same field (×100) of transfected cells. Bottom: GFP-expressing cells with blebbing or normal morphology were counted. The extent of apoptosis was determined by counting GFP-expressing cells with blebbing or normal morphology in three randomly selected fields (80–100 cells per field). The average numbers in three different fields from two independent experiments are shown. Bar, 50 µm.
(TIF)

Figure S3 HeLa cells were transfected with pCS4, pCS4-Myc-Rad9-wt, or pCS4-Myc-Rad9-S328A. Equal amounts of protein from mitochondrial fractions were resolved by SDS-PAGE and analyzed by immunoblotting using antibodies against Rad9 and COX IV.
(TIF)
