# Supplementary figures and images for: Phosphorylation of Rad9 at Serine 328 by Cyclin A-Cdk2 Triggers Apoptosis via Interfering Bcl-xL
Source: PLoS One. 2012 Sep 13;7(9):e44923. doi: 10.1371/journal.pone.0044923 (PMC3441668; doi:10.1371/journal.pone.0044923)

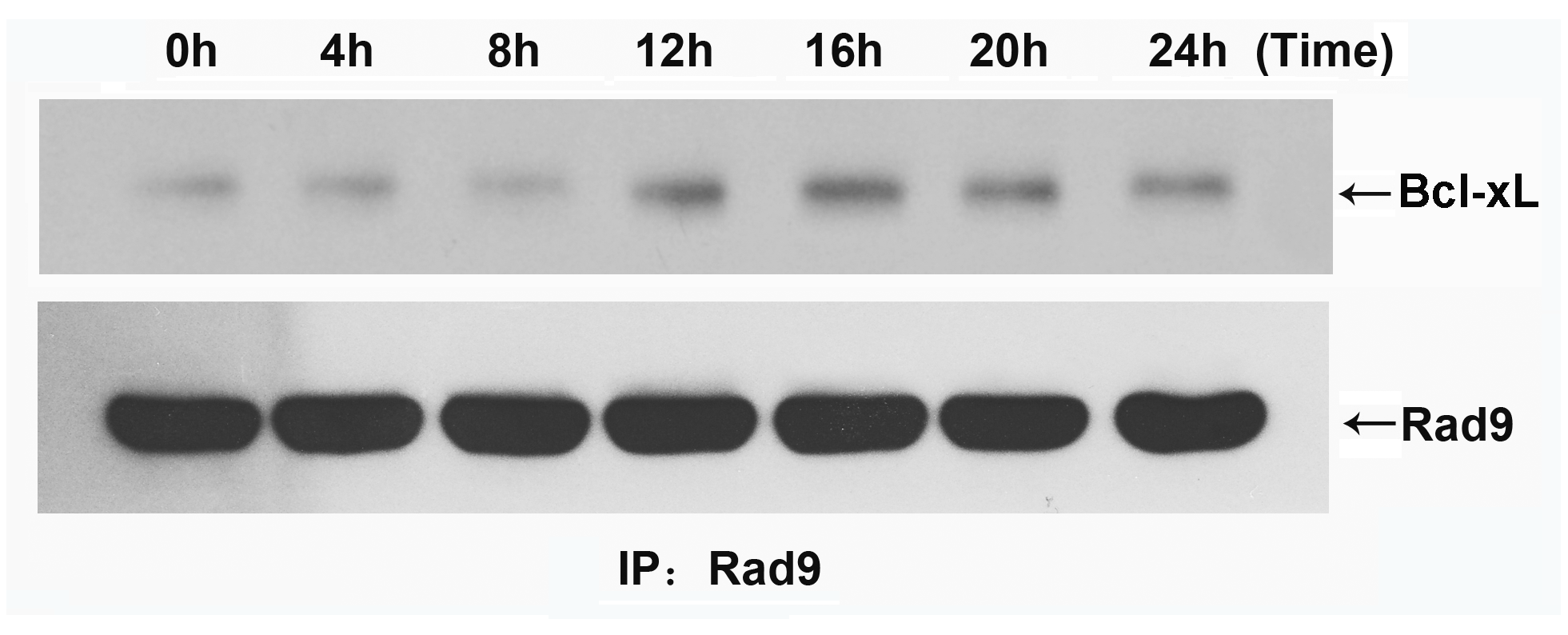

Supplement: Figure S1 — HeLa cells were treated with etoposide (50 µg/mL) for the indicated times. Lysates from treated cells were subjected to immunoprecipitation with anti-Rad9 antibody and immunoblotting with antibodies against Bcl-xL and Rad9. (TIF) [file pone.0044923.s001.tif]

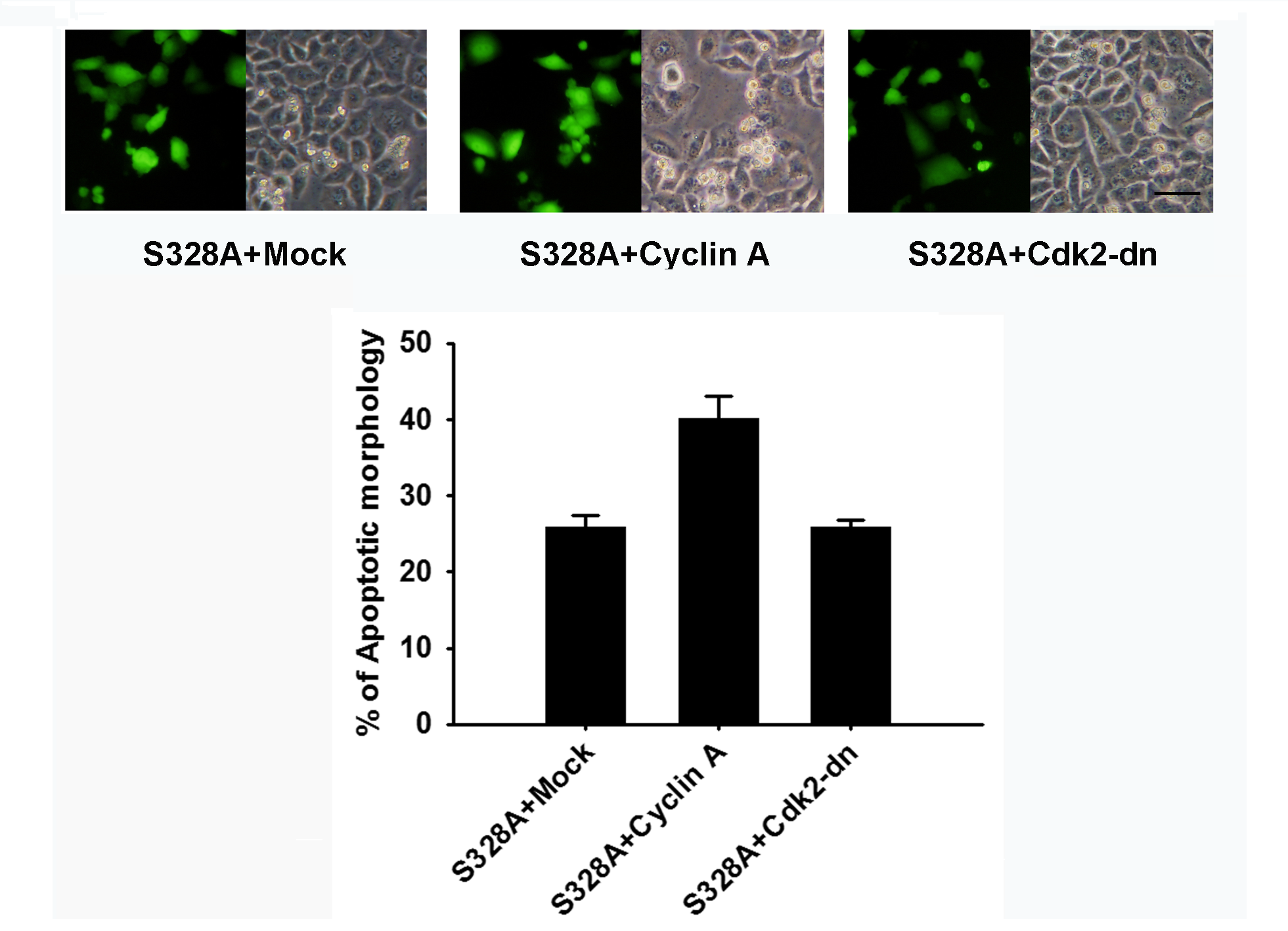

Supplement: Figure S2 — HeLa cells were co-transfected with pCMV-GFP, pCS4-myc-Rad9-S328A, pCMV-Cyclin A, or pCMV-Cdk2-dn. Top: Bright-field and GFP fluorescence of the same field (×100) of transfected cells. Bottom: GFP-expressing cells with blebbing or normal morphology were counted. The extent of apoptosis was determined by counting GFP-expressing cells with blebbing or normal morphology in three randomly selected fields (80–100 cells per field). The average numbers in three different fields from two independent experiments are shown. Bar, 50 µm. (TIF) [file pone.0044923.s002.tif]

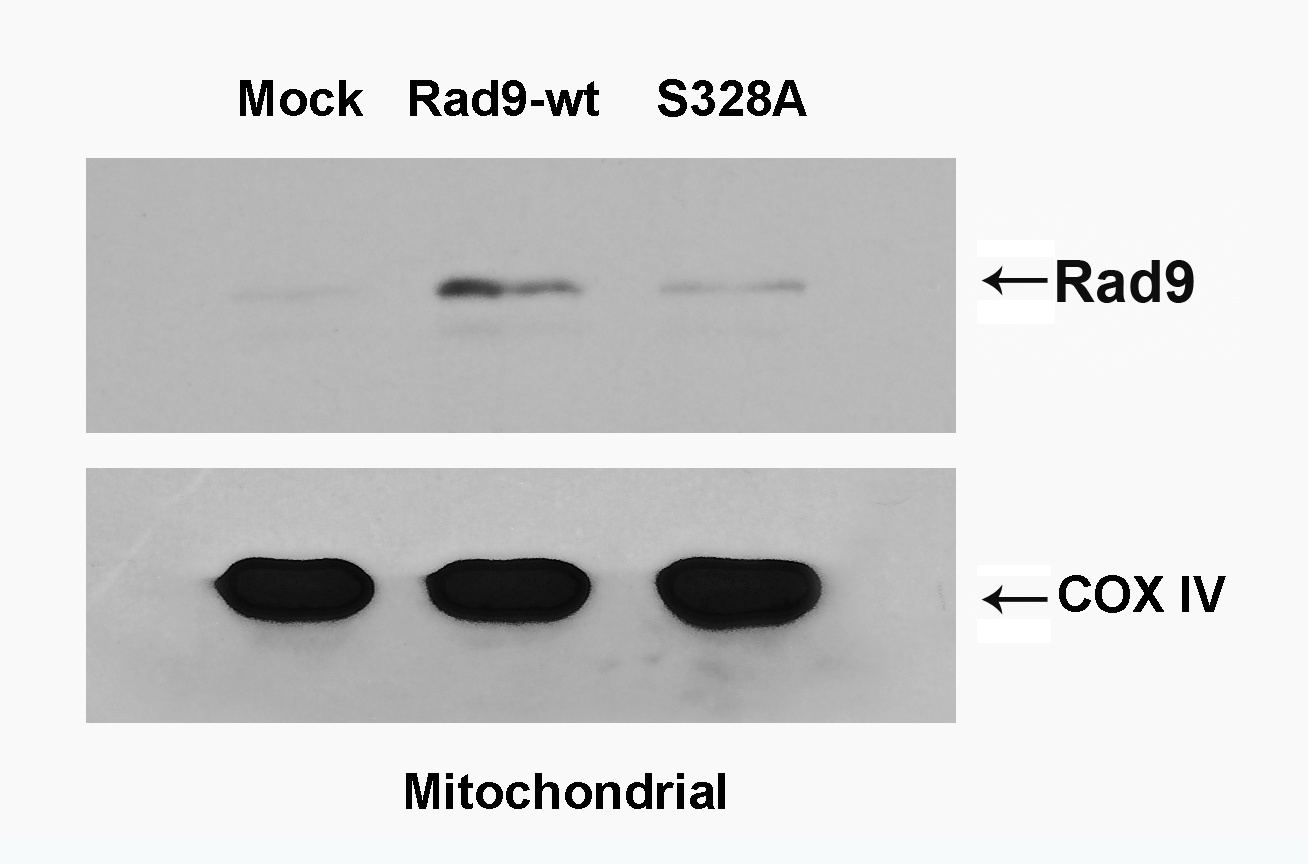

Supplement: Figure S3 — HeLa cells were transfected with pCS4, pCS4-Myc-Rad9-wt, or pCS4-Myc-Rad9-S328A. Equal amounts of protein from mitochondrial fractions were resolved by SDS-PAGE and analyzed by immunoblotting using antibodies against Rad9 and COX IV. (TIF) [file pone.0044923.s003.tif]
